# An Empirical Study of the Impact of Social Interaction on Public Pro-Environmental Behavior

**DOI:** 10.3390/ijerph16224405

**Published:** 2019-11-11

**Authors:** Junjun Zheng, Mingmiao Yang, Mingyuan Xu, Cheng Zhao, Cong Shao

**Affiliations:** Economics and Management School, Wuhan University, Wuhan 430072, China; zhengjunjun@whu.edu.cn (J.Z.); mingmiao.yang@whu.edu.cn (M.Y.); 2018201050198@whu.edu.cn (C.Z.); 2015201050192@whu.edu.cn (C.S.)

**Keywords:** social interaction, public pro-environmental behavior, OLS regression, empirical research

## Abstract

Public pro-environmental behavior plays a positive role in solving environmental pollution problems. In a real socioeconomic system, because public pro-environmental behavior has characteristics of externality and interactivity, a number of factors, such as external information and the behavior of others, could affect the pro-environmental behavior of individuals who optimize their own strategies by interacting with the outside world; thus, public pro-environmental behavior and social interaction are very closely related. In order to study the impact of social interaction on public pro-environmental behavior and its mechanisms, the authors of this paper conducted an empirical study based on an Ordinary Least Square (OLS) regression model and data from the Chinese General Social Survey (CGSS). The empirical results show that: (1) social interaction has a promoting effect on public environmental protection behavior, and social interaction has a more significant impact on private environmental protection behavior; (2) the public will not only adjust their own environmental protection behavior by directly observing the behavior of others, they will also obtain environmental protection knowledge through social interactions which thus have a positive impact on their behavior. It is of great practical significance to study the impact of social interactions on public pro-environmental behavior.

## 1. Introduction

With the continuous advancement of the industrialization process, the economy of China has rapidly developed and the living standards of people have also been greatly improved. At the same time, environmental problems have become increasingly prominent. Environmental pollution not only has a negative impact on the physical and mental health of the public, it also is not conducive to the sustainable development of society and the economy. In this context, the report of the 17th National Congress of the Communist Party of China (CPC) put forward the concept of “ecological problems.” After that, the report of the CPC 19th National Congress incorporated ecological civilization into the modernization system of China and focused on a new pattern of promoting the harmonious development of man and nature. However, to solve the problem of environmental pollution, we should rely not only on the administrative support of the government but also the active support and participation of the general public. In a real socio-economic system, in addition to transmitting signals to the outside world, the public also receives information from the external environment, thereby the social interaction among individuals is created. Based on the theory of social influence effects, some factors such as information from outside world and the behavioral choices of others may have an impact on individual behavior [1,2].Therefore, it is of great practical significance to study the impact of social interactions on public pro-environmental behavior.

“Relationship” culture has been deeply rooted in China for a long time, and it has played a lubricating role in the process of social development in our country. In this social context, the impact of social interaction on public behavior choices cannot be ignored. Rao et al. [3] found that the social interactions of family members have a positive effect on family lending behavior. Liu et al. [4] found that online social interaction can promote family security investment and real estate investment participation, and its promoting effect is higher than that in offline social interaction. Guo et al. [5] believed that social interaction can increase the stock participation of family members by transmitting capital market information. In environmental issues, social interaction is also crucial. Zhang et al. [6] believed that the mass media can promote public pro-environmental behavior through information dissemination and social mobilization mechanisms. In other words, the public can obtain environmental information and environmental knowledge through the mass media and then take appropriate environmental behavior.

The existing research on public pro-environmental behavior have achieved relatively fruitful research results, many scholars have used social surveys, empirical research and other methods to study the impact of one or more factors on public pro-environmental behavior. For example, Zheng et al. [7] studied the effects of responsibility attribution, consequences consciousness, and ethical principles on public pro-environmental behavior, but research on information interaction in the process of behavior selection has been neglected. However, in real life, the public can not only obtain environmental information through the mass media but also gain environmental protection knowledge in the social processes with neighbors and friends. At the same time, the behavior of their neighbors and friends may also affect their own environmental behavior. Because of the external and interactive characteristics of environmental behavior in real life, the choice of public environmental strategy is closely related to the information people own. When individuals make environmental decisions, they interact with the outside world to obtain information, including environmental knowledge and environmental behaviors of others, in order to optimize their strategic choices.

Based on above, the authors of this paper use the data from the Chinese General Social Survey (CGSS) to deeply explore the impact of social interaction on public pro-environmental behavior and the mechanisms of how social interactions influence public pro-environmental behavior through information. Firstly, according to the study of Wang et al. [8], public pro-environmental behavior can be divided into private pro-environmental behavior and common pro-environmental behavior. Because there are some differences in the pro-environmental behavior involved between the two, there may be some differences in the impacts of social interaction on the two types of behavior. Therefore, the pro-environmental behaviors involved in the questionnaire were divided into private pro-environmental behaviors and common pro-environmental behaviors, and then relevant empirical research was carried out. Secondly, in order to explore the mechanisms of how social interactions influence public pro-environmental behavior through information, the authors of this paper deconstructed the impact of social interaction on public pro-environmental behavior and presents a correlation analysis by drawing on the conduction path analysis method proposed by Mo [9]. Compared with previous studies, our study makes an attempt to explore the impact of social interactions on public pro-environmental behavior using empirical methods.

The rest of this paper is organized as follows. In Section 2, relevant literatures are introduced and research hypotheses are proposed. In Section 3, empirical research is designed. In Section 4, the empirical results are analyzed. The last section draws conclusions.

## 2. Relevant Literature and Research Hypotheses

### 2.1. Social Interaction, Environmental Protection Knowledge and Public Pro-Environmental Behavior

The acquisition of information related to environmental protection has a crucial influence on the choice of individual environmental protection behavior. Generally, we can divide environmental protection information into two aspects: environmental knowledge and the environmental behavior of others. In real life, the information that individuals possess is incomplete, which may result in individual decisions failing to achieve expected results. Therefore, individuals need to interact with the outside world to obtain information to optimize their decisions.

However, social interaction is complex—based on traditional economic theory, Manski [10] divided it into three levels: endogenous interactions, contextual interactions, and correlated effects. Among them, an endogenous interaction refers to the relationship between individual behavior and group behavior. On the one hand, individual behavior is affected by group behavior; on the other hand, individual behavior, in turn, affects group behavior. The mechanisms of the two kind of impacts include obtaining information and social norms through interactions. Contextual interaction means that individual behavior is affected by certain group characteristics that are exogenous to the group. However, this kind effect is unilateral, which is unlike endogenous interactions. The correlated effects suggest that certain similar behaviors of individuals in the same group may be related to the similarities in the environment in which they live [5]. Ellison et al. [11] argued that when individuals are unable to accurately assess the costs and benefits of a particular strategy choice, they often use verbal communication to obtain information and help them make decisions. Since then, Brown et al. [12] and Zhou et al. [13] have verified the view of Ellison et al. [11] through empirical research. In fact, social interaction provides individuals with a channel for information acquisition so that individuals can observe and learn [14]. In the process of selecting environmental protection strategies, individuals can obtain environmental protection knowledge and other people’s environmental behavior through social interaction and then adjust their strategies [10]. Therefore, this article mainly explores the relationship between endogenous interactions and public pro-environmental behavior.

### 2.2. Additional Relevant Factors and Public Pro-Environmental Behavior

#### 2.2.1. The Degree of Environmental Pollution

It is generally believed that the higher the degree of environmental pollution, the greater the public’s willingness to protect the environment, and the higher the probability of adopting environmental protection behavior. For example, Inglehart [15] found that public environmental protection willingness is stronger in areas with relatively serious environmental pollution than in areas with relatively light environmental pollution; Wang et al. [8] analyzed the data from the CGSS (2013) and found that public environmental pollution perception has a positive impact on environmental behavior, and the impact of environmental pollution on environmental behavior is affected by the level of economic development. However, some scholars believe that there is a difference between objective environmental pollution and the public perception of pollution. Many pollutions are not perceived by the public, and it is thus difficult to have a substantial impact on public pro-environmental behavior [16]. In fact, some scholars have proven this point through empirical research. For example, Franzen et al. [17] found that there is no significant correlation between environmental quality and individual environmental attitudes.

#### 2.2.2. Income Level

By using empirical research, Hadler et al. [18] found that individuals’ private environmental behaviors and public pro-environmental behaviors are affected by household income. The higher the income, the higher the probability of choosing private environmental behavior, and the lower the probability of choosing public pro-environmental behaviors. Tindall et al. [19] found that income has an impact on environmental behavior. Wang Yujun et al. [8] also found that income has a positive impact on individual environmental behavior.

#### 2.2.3. Education Level

At present, a large number of studies have found that there is a positive correlation between education level and environmental behavior. For example, Callan et al. [20] found that individuals with a higher level of education were more likely to recycle resources. Monier et al. [21] found a positive correlation between education and green food purchases. Meyer [22] also found that education can promote environmental behavior. In fact, education can influence individual environmental behaviors at different levels: Firstly, education can improve the environmental knowledge level of individuals, while a higher level of knowledge makes individuals have a stronger ability to identify environmental problems and more likely to take relevant environmental protection measures; secondly, education can subtly enhance individual environmental awareness and then make individuals pay more attention to environmental issues; thirdly, individuals with higher education tend to have a higher income, and there is a positive correlation between income level and environmental protection behavior.

#### 2.2.4. Gender

Many scholars have found that there are gender differences in environmental behaviors, that is women have a stronger awareness of environmental protection and a stronger motivation to do pro-environmental behaviors than men [8,23,24,25]. There are two possible explanations for this: one explanation is that gender as a mediator can regulate sustainable consumer behavior. For example, because of differences in personality traits, female consumers are more concerned with social and environmental issues [24]. Another explanation is that women have a higher level of socialization and social responsibility than men, and this may have an impact on individual environmental behavior [26].

### 2.3. Research Hypothesis

Based on the above analysis, we believe that the mechanism of how social interaction influences public pro-environmental behavior through information mainly involves two aspects:

Firstly, the public obtains environmental protection knowledge through social interactions and thus influences their behavior choices. Hadler et al. [18] found a positive correlation between environmental knowledge and public pro-environmental behavior through empirical research. Wang Yujun et al. [8] studied the environmental behaviors of Chinese residents and found that environmental knowledge has a positive effect on private environmental behaviors and public environmental behaviors. Pothitou et al. [27] found that environmental knowledge has a positive impact on household environmental attitudes and environmental behaviors.

Secondly, the public determine their own strategies by directly observing the behavior of others.

Thus, the authors of this paper posit the following hypotheses:

**Hypothesis** **1 (H1).** *Social interaction can promote public pro-environmental behavior.*

**Hypothesis** **2 (H2).** *There is a positive correlation between environmental knowledge and environmental behavior held by the public, which means that environmental knowledge promotse environmental behavior.*

## 3. Research Design

### 3.1. Data Source

The data selected in this paper were derived from the China Comprehensive Social Survey (CGSS2013), which was jointly conducted by Renmin University of China and relevant academic institutions. The questionnaire is mainly composed of social population attributes, health, lifestyle, social attitudes, and personal cognitive abilities. The data were collected by face-to-face interviewing. The total sample number obtained through the survey was 11438. After eliminating the invalid and default data, the effective sample size number was 7472.

### 3.2. Variable Description

#### 3.2.1. Dependent Variable

The dependent variable for this paper was public pro-environmental behavior, and the data were taken from the environmental aspects of the 2013 CGSS questionnaire. The CGSS investigated public pro-environmental behavior through 10 questions, such as garbage sorting, discussing environmental issues with friends and relatives, and preparing baskets or bags when shopping. The options available to respondents were “never,” “occasionally,” and “frequently,” with assignments of 0, 1 and 2, respectively. The questions are shown in Table 1.

First of all, the authors analyzed public pro-environmental behavior from the overall level. The authors summed up the scores of each respondent on 10 questions and calculates the score of public pro-environmental behavior, which means that the lower the score, the less environmental action the respondent takes. The function is shown as below.

(1) $$Score_{i}=\sum_{j}EP_{ij}$$

In the function, $EP_{ij}$ represents the score of respondent i on question j.

Secondly, following to the analysis results of Wang et al. [8], the pro-environmental behavior corresponding to the 10 questions was divided into private pro-environmental behavior and common pro-environmental behavior in this paper. Private pro-environmental behavior refers to the behavior of someone purchasing, using or cleaning personal and household items that may have an impact on the environment; common pro-environmental behavior mainly refers to the behavior of someone supporting or accepting public environmental policies. Therefore, Questions 1–4 and 6 belonged to private pro-environmental behavior, and Question 5 and 7–10 belonged to common pro-environmental behavior. The score functions of private pro-environmental behavior and common pro-environmental behavior of the respondent i are shown, respectively, as follows:

(2) $$Score_{i}^{private}=\sum_{j=1}^{4}EP_{ij}+EP_{i6}$$

(3) $$Score_{i}^{common}=EP_{i5}+\sum_{j=7}^{10}EP_{ij}$$

Based on above, in order to further explore the relationship between public pro-environmental behavior and social interaction, the statistics of public pro-environmental behavior re shown in Table 1.

#### 3.2.2. Independent Variable

The independent variables in this paper mainly included social interaction, environmental knowledge, and additional related variables.

1. Social Interaction

The authors divided the questions about social interaction in the questionnaire into two categories: the objective social interaction behavior and the respondents’ subjective feelings about their social interaction.

The question related to the objective social interaction behavior was “the frequency of social entertainment activities with other friends.” The options available to the respondents were “almost every day,” “1–2 days a week,” “a few times a month,” “about once a month,” “a few times a year,” “one time or less a year,” and “never,” and the corresponding assignments were 1, 2, 3, 4, 5, 6 and 7, respectively. The smaller the assignment was, the higher the social frequency of the respondents was.

The question related to the subjective feelings of respondents was “the situation about contacting with family and friends.” The options available to the respondents are “very not close,” “not close,” “general,” “close,” and “very close,” and the corresponding assignments were 1, 2, 3, 4 and 5, respectively. The larger the assignment was, the closer the social connection that respondents subjectively perceive was.

2. Environmental Knowledge

The questionnaire tested the public’s knowledge of environmental protection through 10 questions. If the answer was correct, the respondent could get 1 point. If the answer was wrong or unknown, the score was 0. The scores of the 10 questions were added to get the comprehensive environmental knowledge score of the respondent. The highest score was 10, and the lowest was 0.

3. Additional Related Variables

The questionnaire on the degree of environmental pollution was mainly designed with 12 aspects, including air pollution, water pollution, noise pollution, domestic garbage pollution, and forest vegetation damage. Due to the relative shortage of knowledge of the public on problems such as the destruction of forest vegetation, the degradation of cultivated land quality, and the shortage of fresh water resources, the data integrity was low. Thus, the authors selected three types of environmental problems as representatives: air pollution, water pollution and domestic garbage pollution. For these environmental problems, the options available to respondents were “very serious,” “quite serious,” “not too serious,” “not serious,” “general,” “not concerned/unclear,” and “no such problem,” and the assignments were 1, 2, 3, 4, 5, 6 and 7, respectively. The smaller the assignment was, the more serious the environmental problem was.

The individual characteristic involved in this paper included gender (male and female), years of schooling, personal income and regions (east, central, west, and northeast—the regions where the respondents located were divided by the method of the national statistics bureau, so the eastern region included Beijing, Tianjin, Hebei, Shanghai, Jiangsu, Zhejiang, Fujian, Shandong, Guangdong and Hainan; the central region included Shanxi, Anhui, Jiangxi, Henan, Hubei and Hunan; the western region included Inner Mongolia, Guangxi, Chongqing, Sichuan, Guizhou, Yunnan, Tibet, Shanxi, Gansu, Qinghai, Ningxia and Xinjiang; and the northeast region included Liaoning, Jilin and Heilongjiang), in which personal income was the logarithm in the empirical analysis process.

### 3.3. Empirical Model

Public pro-environmental behavior was a continuous base, and had many values. Meanwhile the higher the score, the more environmental actions the respondent took.

According to the analysis of Ferreri-Carbonell et al. [28], as long as a model is properly processed, the direction and significance of a model parameters obtained by Ordinary Least Square (OLS) regression and logit regression or probit regression are the same and there is no obvious advantage or disadvantage between them. Thus, the authors of this paper selected OLS regression to analyze the impact of social interaction on public pro-environmental behavior. The empirical model is shown as follows:

(4) $$S_{i}=\beta_{0}+\beta_{1}C_{i}+\beta_{2}K_{i}+\beta_{3}I_{i}+\varepsilon_{i}$$

(5) $$S_{i}^{private}=\beta_{0}+\beta_{1}C_{i}+\beta_{2}K_{i}+\beta_{3}I_{i}+\varepsilon_{i}$$

(6) $$S_{i}^{common}=\beta_{0}+\beta_{1}C_{i}+\beta_{2}K_{i}+\beta_{3}I_{i}+\varepsilon_{i}$$

In these functions, the variables $S_{i}$, $S_{i}^{private}$ and $S_{i}^{common}$ represent public pro-environmental behavior, private pro-environmental behavior, and common pro-environmental behavior, respectively.

$C_{i}$ represents the social interaction behavior of individual i.

$K_{i}$ represents the environmental knowledge possessed by individual i.

$I_{i}$ represents additional relevant variables.

## 4. Analysis of Empirical Results

The descriptive statistics for the variables are shown in Table 2 and Table 3 (the nominal variables are in Table 3). According to the statistical results, the mean value of public pro-environmental behavior was 5.48, the mean value of private pro-environmental behavior was 4.49, and the mean value of common pro-environmental behavior was 0.99, which was significantly lower than private pro-environmental behavior. At the same time, compared with the results of the 2003 survey, the private pro-environmental behavior of Chinese residents has improved in 2013, while common pro-environmental protection behavior has declined [8].

According to the statistical results in Table 2, the mean value of air pollution was 3.43, the mean value of water pollution was 3.52, and the mean value of domestic garbage pollution was 3.46. Overall, the mean value of environmental pollution in China was between “not too serious” and “not serious;” respondents’ average years of schooling was 9.27, which was roughly equal to the junior high school graduation level; the respondents’ overall income level was not high, and the mean value of personal income was about 4.22.

Furthermore, the mean value of environmental knowledge held by respondents was 5.12.

### 4.1. The Impact of Social Interaction on Public Pro-Environmental Behavior

The authors of this paper firstly studied the impact of social interaction on public pro-environmental behavior. In order to enhance the robustness of the regression model, the authors of this paper included additional relevant variables in all three models. Through collinear diagnostic analysis, it could be seen that there was no multicollinearity among the variables selected in this paper. Table 4 shows the corresponding empirical analysis results.

The empirical results showed that social interaction had a positive effect on public pro-environmental behaviors. The higher the frequency of social interaction with friends was and the closer the relationship with friends and relatives was, the more likely one was to adopt environmental protection behavior. At the same time, compared with social frequency, the closeness of contact with relatives and friends had a deeper impact on public pro-environmental behavior, which indicates that the public’s subjective feelings about social interaction can have a more practical impact on their behavior. In fact, it is not difficult to understand that it is often easier for people to accept the opinions of relatives and friends who are more intimate with them in order to learn and imitate their behavior in daily interactions. Consistent with previous studies, the empirical results of this paper showed that the impact of environmental protection knowledge on public pro-environmental behavior was significantly positive; that is the richer the environmental knowledge of individuals was, the more likely they were to participate in environmental protection activities.

Among the additional relevant variables, gender, education years, and personal income had significant effects on public pro-environmental behavior. Women were shown to be more likely to adopt environmental behavior than men. The increase in years of education has contributed to public pro-environmental behavior. At the same time, the growth of personal income has also shown a positive impact on public pro-environmental behavior. Compared with the northeast region, the public pro-environmental behavior indexes of the eastern and the western regions were relatively high, while the difference between the central and the northeast regions was not significant.

In addition, there was a positive correlation between air pollution and public pro-environmental behavior, and the impacts of water pollution and domestic garbage pollution on public pro-environmental behavior were not significant. We believe that this is related to the public’s perception to pollution. In recent years, with the outbreak of smog, the public’s attention to air pollution has continued to rise, and the problem has become a focus of public attention. Compared with air pollution, water pollution and domestic garbage pollution are less concerning, and many people are not fully aware of such pollution problems. As Hyslop [16] said, if pollution is not perceived by the public, environmental pollution is unlikely to have a substantial impact on public pro-environmental behavior.

### 4.2. The Impact of Social Interaction on Private and Common Pro-Environmental Behavior

The authors of this paper divided public pro-environmental behavior into private pro-environmental behavior and common pro-environmental behaviors and then conducted regression analyses separately. The empirical results are shown in Table 5 and Table 6.

In the third model of private environmental behavior, social frequency had no significant impact on private pro-environmental behavior, and relationships with relatives and friends still contributed to private pro-environmental behavior. In the sixth model of common pro-environmental behavior, social frequency had a positive impact on common pro-environmental behavior, and relationships with relatives and friends had no significant impact on common pro-environmental behavior. Generally, the impact of social interaction on private environmental behavior was relatively more significant, which may be related to the low participation of the public in common environmental protection activities. The impact of environmental protection knowledge on private environmental behavior and common pro-environmental behaviors was significantly positive, but its impact on private pro-environmental behavior was higher than common pro-environmental behavior.

Among the additional relevant variables, the impact of schooling years and personal income on private and common pro-environmental behaviors remained significant, and there are no more details here. In the private pro-environmental behavior model, the participation of women was significantly higher than that of men. In the common pro-environmental behavior model, the gender impact was not significant, which is different from previous research conclusions.

Furthermore, among the environmental pollution variables, there was a significant correlation between water pollution and common pro-environmental behavior. The more serious the water pollution was, the higher the public participation in common pro-environmental activities was. We believe that this may be related to the character of water pollution control methods. Since water pollution control often requires the participation of government departments, when such pollution problems arise, the public may adopt more public environmental protection measures such as complaints to environmental protection departments.

### 4.3. Research on the Conduction Path of Social Interaction to Public Pro-Environmental Behavior

The empirical results in the previous article showed that social interaction can indeed affect public pro-environmental behavior, and its impact on private pro-environmental behavior is different from common pro-environmental behavior. If so, how does it affect public pro-environmental behavior? Thus, we used the conduction path analysis method proposed by Mo [9] to deconstruct the impact of social interaction on public pro-environmental behavior. The function is shown as below:

(7) $$\frac{dS}{dC}=\frac{\partial S}{\partial C}+\frac{\partial S}{\partial K}\cdot \frac{\partial K}{\partial C}$$

where S represents public pro-environmental behavior, C represents social interaction, K represents environmental knowledge, $\frac{\partial S}{\partial C}$ depicts the direct impact of social interaction on public pro-environmental behavior, and $\frac{\partial S}{\partial K}\cdot \frac{\partial K}{\partial C}$ depicts the indirect impact of social interaction on public pro-environmental behavior through environmental knowledge, of which the sum is the comprehensive effect of social interaction on public pro-environmental behavior.

In this paper, environmental knowledge was considered as the dependent variable, and social interaction was the independent variable. A correlation analysis was carried out, and the results are shown in Table 7.

**Figure 1:**
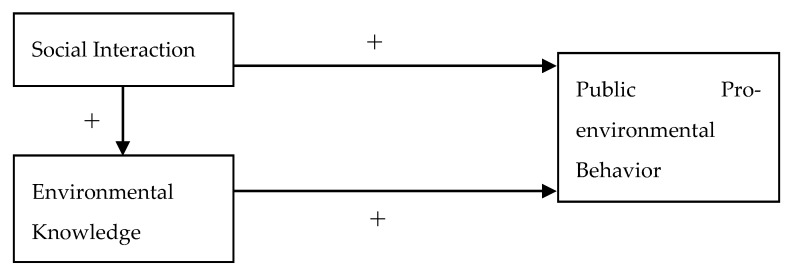
Conduction path.

The correlation analysis results show that there was a negative correlation between social frequency and environmental knowledge, and there was a positive correlation between the degree of closeness and environmental knowledge. That is to say, the higher the social frequency was and the closer the relationship with relatives and friends was, the higher the environmental knowledge was. According to the empirical results mentioned above, there was a positive correlation between environmental knowledge and public pro-environmental behavior, which indicates that social interaction can improve public environmental knowledge level through an information transmission mechanis, and promote public pro-environmental behavior. Therefore, we can obtain the conduction path diagram as shown in Figure 1.

## 5. Conclusions

Based on the data of the CGSS 2013, the authors of this paper analyzed the influence of social interaction on public pro-environmental behavior and its mechanisms. Public pro-environmental behavior was analyzed at first, and the empirical results showed that social interactions have a positive effect on such behavior, which validates previous studies [8,15,16,17] and makes new progress by identifying the impact mode. Furthermore, the following conclusions have been obtained:

1. At the individual level, the environmental protection participation of females is higher than that of males, which is in accordance with previous studies [8,23,24,25], and there is a positive correlation among education degree, personal income, environment knowledge, and public pro-environment behavior, which is also is in accordance with previous studies [8,18,19,20,21,22].
2. Public pro-environmental behavior is divided into private and common pro-environmental behavior, and the descriptive statistics showed that public participation in the field of private environmental protection is much higher than that of common environmental protection. The results of the regression analysis showed that the effect of social interaction on private pro-environmental behavior is relatively more significant, which is a new finding.
3. Using a conduction path analysis method to deconstruct the impact of social interaction on public pro-environmental behavior, the authors were able to find that: (1) The public adjusts their pro-environmental behavior by directly observing the behavior of others; (2) the public acquires environmental knowledge through social interaction, and then the level of environmental knowledge can further influence their behavior.

Based on the above conclusions, we suggest that in order to protect the environment, the government should promote social interaction and pay more attention to the environmental protection participation of males. Otherwise, the government ought to guide the masses to increase their common environmental protection. Last but not least, the publicity of environmental knowledge is of vital importance.

One research limitation is that some of the used variables used were somewhat general. In future studies, they can be divided into more specific variables. For example, the variable of the degree of closeness with relatives and friends contained at least two aspects, so it can be divided into the two specific variables: the degree of closeness with relatives and the degree of closeness with friends. Future studies can attempt to find out how the two specific variables effect pro-environmental behavior if the data are available. It will be interesting if future studies found that the two specific variables may have different effects on pro-environmental behavior.

## Figures and Tables

**Table 1:** Statistics of public pro-environmental behavior.

NO.	Activity or Behavior	Never	Occasionally	Often
1	Garbage sorting	52.9%	34.1%	12.9%
2	Discuss environmental issues with your friends and relatives	45.7%	45.2%	9.2%
3	Bring your own shopping basket or shopping bag when purchasing daily necessities	22.1%	35.6%	42.3%
4	Reuse plastic packaging	16.9%	30.8%	52.2%
5	Donate for environmental protection	81.8%	16.3%	1.9%
6	Actively focus on environmental issues reported in radio, television and newspapers and environmental information	45.1%	39.8%	15.2%
7	Actively participate in environmental publicity and education activities organized by the government and organizations	82.6%	14.6%	2.8%
8	Actively participate in environmental protection activities organized by private environmental groups	82.6%	14.6%	2.8%
9	Self-funded forest or green space	85.2%	11.0%	3.8%
10	Actively participate in complaints and appeals that require environmental problems	90.7%	7.8%	1.4%

**Table 2:** Descriptive statistics of variables.

	Mean Value	Standard Deviation	Maximum Value	Minimum Value
Public pro-environmental behavior	5.48	3.33	20	0
Private environmental behavior	4.49	2.34	10	0
Common pro-environmental behavior	0.99	1.62	10	0
Social frequency	3.83	1.72	7	1
Degree of closeness with relatives and friends	3.47	0.84	5	1
Years of schooling	9.27	4.28	19	0
Personal income	4.22	0.49	6	1.9
Air pollution	3.43	1.89	7	1
Water pollution	3.52	1.88	7	1
Domestic garbage	3.46	1.77	7	1
**Environmental knowledge**	5.12	2.71	10	0

**Table 3:** Descriptive statistics of nominal variables.

Variable	Value	Frequency
Gender	Male	56.0%
	Female	44.0%
Region	East	36.7%
	Central	24.4%
	West	25.5%
	Northeast	13.4%
Political countenance	Communist	12.7%
	Democratic parties	0.1%
	Communist youth league member	3.7%
	Common people	83.5

**Table 4:** Impact of social interaction on public pro-environmental behavior.

	Model 1	Model 2	Model 3
Social frequency	−0.103 ***		−0.085 ***
	(0.000)		(0.000)
Degree of closeness with relatives and friends		0.203 ***	0.163 ***
		(0.000)	(0.000)
Female	0.634 ***	0.609 ***	0.615 ***
	(0.000)	(0.000)	(0.000)
Years of schooling	0.159 ***	0.156 ***	0.156 ***
	(0.000)	(0.000)	(0.000)
Personal income	0.532 ***	0.524 ***	0.520 ***
	(0.000)	(0.000)	
Region: Taking the northeast region as a reference group			
East	1.053 ***	1.048 ***	1.051 ***
	(0.000)	(0.000)	(0.000)
Central	0.057	0.092	0.067
	(0.622)	(0.427)	(0.564)
West	0.498 *	0.482 *	0.488 **
	(0.065)	(0.063)	(0.042)
Air pollution	−0.134 ***	−0.138 ***	−0.135 ***
	(0.000)	(0.000)	(0.000)
Water pollution	0.000	−0.002	−0.004
	(0.990)	(0.928)	(0.876)
Domestic garbage	−0.008	−0.005	−0.007
	(0.712)	(0.816)	(0.750)
Environmental knowledge	0.234 ***	0.203 ***	0.233 ***
	(0.000)	(0.000)	(0.000)
Number of jobs	7472	7472	7472
R-squared	0.211	0.212	0.213
Adj R-squared	0.210	0.210	0.211

*p* values are shown in parentheses, and *, **, and *** indicate significant levels of significance at 10%, 5%, and 1%, respectively.

**Table 5:** Impact of social interaction on private pro-environmental behavior.

	Model 1	Model 2	Model 3
Social frequency	−0.034 **		−0.016
	(0.017)		(0.286)
Degree of closeness with relatives and friends		0.172 ***	0.165 ***
		(0.000)	(0.000)
Female	0.621 ***	0.602 ***	0.603 ***
	(0.000)	(0.000)	(0.000)
Years of schooling	0.114 ***	0.112 ***	0.112 ***
	(0.000)	(0.000)	(0.000)
Personal income	0.525 ***	0.512 ***	0.512 ***
	(0.000)	(0.000)	(0.000)
Air pollution	−0.138 ***	−0.140 ***	−0.139 ***
	(0.000)	(0.000)	(0.000)
Water pollution	0.025	0.022	0.022
	(0.166)	(0.229)	(0.237)
Domestic garbage	−0.002	0.000	−0.001
	(0.913)	(0.985)	(0.968)
Environmental knowledge	0.202 ***	0.202 ***	0.201 ***
	(0.000)	(0.000)	(0.000)
Number of jobs	7472	7472	7472
R-squared	0.206	0.210	0.210
Adj R-squared	0.206	0.209	0.209

*p* values are shown in parentheses, and *, **, and *** indicate significant levels of significance at 10%, 5%, and 1%, respectively.

**Table 7:** Results of correlation analysis between social interaction and environmental knowledge.

	Social Frequency	Degree of Closeness with Relatives and Friends
**Estimate of coefficient**	−0.200	0.322
***t*-value**	−11.013	8.626

**Table 6:** Impact of social interaction on common pro-environmental behavior.

	Model 4	Model 5	Model 6
Social frequency	−0.060 ***		−0.057 ***
	(0.000)		(0.000)
Degree of closeness with relatives and friends		0.051 **	0.024
		(0.020)	(0.282)
Female	0.020	0.013	0.017
	(0.590)	(0.732)	(0.639)
Years of schooling	0.070 ***	0.069 ***	0.069 ***
	(0.000)	(0.000)	(0.000)
Personal income	0.213 ***	0.213 ***	0.211 ***
	(0.000)	(0.000)	(0.000)
Air pollution	−0.026 *	−0.028 **	−0.027 *
	(0.053)	(0.040)	(0.052)
Water pollution	−0.029 **	−0.028 **	−0.029 **
	(0.035)	(0.040)	(0.032)
Domestic garbage	0.005	0.007	0.005
	(0.653)	(0.573)	(0.644)
Environmental knowledge	0.039 ***	0.041 ***	0.039 ***
	(0.000)	(0.000)	(0.000)
Number of jobs	7472	7472	7472
R-squared	0.084	0.081	0.084
Adj R-squared	0.083	0.080	0.083

*p* values are shown in parentheses, and *, **, and *** indicate significant levels of significance at 10%, 5%, and 1%, respectively.
